# *Tuber indicum and T. lijiangense* colonization differentially regulates plant physiological responses and mycorrhizosphere bacterial community of *Castanopsis rockii* seedlings

**DOI:** 10.3389/fpls.2023.1134446

**Published:** 2023-04-12

**Authors:** Lanlan Huang, Yongmei Li, Jing Yuan, Shanping Wan, Carlos Colinas, Xinhua He, Xiaofei Shi, Yanliang Wang, Fuqiang Yu

**Affiliations:** ^1^ College of Resources and Environment, Yunnan Agricultural University, Kunming, China; ^2^ The Germplasm Bank of Wild Species, Yunnan Key Laboratory for Fungal Diversity and Green Development, Kunming Institute of Botany, Chinese Academy of Sciences, Kunming, Yunnan, China; ^3^ Department of Crop and Forest Science, University of Lleida, Lleida, Spain; ^4^ Centre of Excellence for Soil Biology, College of Resources and Environment, and Chongqing Key Laboratory of Plant Resource Conservation and Germplasm Innovation, School of Life Sciences, Southwest University, Chongqing, China; ^5^ School of Biological Sciences, University of Western Australia, Perth, WA, Australia; ^6^ Guizhou Kangqunyuan Biotechnology Co., LTD, Liupanshui, Guizhou, China

**Keywords:** ectomycorrhiza, nutrient acquisition, phoC and phoD, phosphatase activity, rhizosphere exudates, mycorrhizosphere microbiome

## Abstract

Black truffles and white truffles are widely studied around the world, but their effects on plant growth and physiological responses, and on the mycorrhizosphere bacterial community of the host plant remain unclear. Here, mycorrhizal colonization of *Castanopsis rockii* by *Tuber indicum* (Chinese black truffle) and *T. lijiangense* (Chinese white truffle), respectively, was induced in a greenhouse study, and their effects on host growth, physiological responses and mycorrhizosphere bacterial communities were compared. The results show that colonization of both *Tuber* species significantly increased leaf photosynthetic rate, leaf P concentration and mycorrhizosphere acid phosphatase activity, as well as richness of mycorrhizosphere bacterial communities of *C. rockii* seedlings. However, *T. indicum* colonization on the one hand significantly decreased tartrate content, bacterial acid phosphatase, *phoC* gene abundance in the mycorrhizosphere, and peroxidase (POD) activity of ectomycorrhizal root tips, but on the other hand increased mycorrhizosphere pH and superoxide dismutase (SOD) of ectomycorrhizal root tips, compared to *T. lijiangense* colonization. Moreover, principal coordinate and β-diversity analyses show significant differences in mycorrhizosphere bacterial community composition between *T. indicum* and *T. lijiangese* colonized *C. rockii* seedlings. Finally, the relative abundance of the bacterium *Agromyces cerinus* significantly correlated to mycorrhizosphere acid phosphatase activity and leaf P concentration, suggesting that this bacterium might play an important role in P mobilization and acquisition. Overall, these results suggest that *T. indicum* and *T. lijiangense* differently regulate their host plant’s physiological responses and mycorrhizosphere bacterial community.

## Introduction

Ectomycorrhizal (ECM) fungi form mutually beneficial associations with host plant roots, which are characterized by the presence of a fungal mantle that envelops lateral roots and a Hartig net that surrounds root epidermal and/or cortical cells (Smith and Read, 2008). About 6,000 woody plant species of mostly temperate and boreal forests form associations with ectomycorrhizal fungi (van der Heijden et al., 2015). A variety of factors have been found to affect the development of symbiosis, such as the host species, surrounding environment and management practices (Leonardi et al., 2013; Li et al., 2018). Ectomycorrhizae can promote plant growth, and provide resistance to stress and soil pathogens through an enhanced uptake of soil nutrients (Smith and Read, 2008; Cairney, 2011; Nehls and Plassard, 2018). Ectomycorrhizae can also release carbon- (C) and nitrogen-(N) containing exudates, including amino acids, organic acids and enzymes to mobilize less available nutrients from soil (Smith and Read, 2008; Cairney, 2011; Wang and Lambers, 2020; Wang et al., 2021), and alter root carbon exudation and rhizosphere bacterial communities (Wang et al., 2021). Furthermore, ECM fungi colonization can drive changes in rhizosphere bacterial communities thus affect the formation and eco-physiological roles of ectomycorrhizae (Berendsen et al., 2012), as well as host plant performance (Izumi et al., 2006; Bulgarelli et al., 2013).

A group of fungi belonging to the genus *Tuber* (Ascomycota, Pezizales), which produce edible hypogeous ascocarps called truffles (Benucci and Bonito, 2016), are typical ectomycorrhizal fungi that form symbiotic relationships with several genera of trees, such as *Pinus*, *Quercus* and *Castanopsis* (Trappe et al., 2009; Benucci et al., 2012; Marozzi et al., 2017; Freiberg et al., 2021; Huang et al., 2021). The ECM symbiosis spans most of the life cycle of truffles, and the quality of truffle-colonized seedlings early in the symbiosis development is an important factor that determines the production of truffle fruiting bodies in plantations (Andres-Alpuente et al., 2014; Mello and Balestrini, 2018). The genus *Tuber* displays a widely geographic distribution, from North Europe, North Africa, Asia, to North and South America (Bonito et al., 2013; Lancellotti et al., 2016; Le Tacon, 2016). Some *Tuber* species such as *T. melanosporum* and *T. magnatum* have highly economic value because of their specific taste and unique fragrance (Bertini et al., 2006; Mello et al., 2006). *Tuber*-associated host tree species including *Pinus*, *Quercus* and *Populus* have important ecological values as major reforestation species (Liu et al., 2014). Studies have shown that ectomycorrhizae formed by *Tuber* species promote the growth of their host plants (Alvarez-Lafuente et al., 2018; Wang et al., 2021; Huang et al., 2022). *Tuber* ectomycorrhizae can also alter root carbon exudation and metabolic profiles of host plants, as well as mycorrhizosphere microbial communities (Benucci et al., 2016; Deveau et al., 2016; Li et al., 2018; Li et al., 2019; Yang et al., 2019; Marjanović et al., 2020; Wang et al., 2020; Wang et al., 2021; Marozzi et al., 2022).

*Tuber indicum*, known as Chinese black truffle, has been successfully cultivated with *Quercus aliena* in southwest China (Wang et al., 2020). *T. indicum* has similar morphological characteristics and a close phylogenetic relationship with *T. melanosporum* (Murat et al., 2008) and associates with more than 20 tree species belonging to different plant families such as Fagaceae, Juglandaceae, Pinaceae and Salicaceae (Huang et al., 2020; Wang et al., 2020). *T. lijiangense*, known as Chinese white truffle, has been firstly described from a natural *Pinus yunnanensis* forest in Yunnan, southwest China, and its ascomata have a strong pleasant aroma (Fan et al., 2011). Both species are expensive in local markets in southwest China (Liu et al., 2011; Wan et al., 2016). Studies have shown that *T. indicum* could increase soil organic matter in rhizosphere of *Q. aliena*, affect host uptake of phosphorus (P) and N, and alter rhizosphere microbial communities (Li et al., 2018; Zhang et al., 2020). However, little information is available about effects of *T. lijiangense* colonization on the performance of host plants and soil characteristics. Therefore, whether the colonization by *T. indicum* or *T. lijiangense* could have similar or different impacts on host plant growth, physiological responses and rhizosphere microbial community are still largely unknown.

*Castanopsis* species belonging to Fagaceae family are usually large canopy trees, widespread generalists growing in different habitats with varying altitudes and soil types (Dong et al., 2009; Cheuk and Fischer, 2021). Some species in the *Castanopsis* genus are comparatively fast-growing, suitable for controlling soil erosion and reforestation (Ren et al., 2008; Dong et al., 2009; Cheuk and Fischer, 2021). For instance, *Castanopsis rockii*, an endemic woody species and a potential *Tuber* host, is widely utilized as a timber tree, in Yunnan, China. The objectives of the study were to assess whether: 1) both *Tuber* species could form ectomycorrhizae with *C. rockii* under controlled conditions; 2) *T. indicum* and *T. lijiangense* alter plant growth, nutrient uptake and physiological responses in *C. rockii* mycorrhizosphere; and 3) *T. indicum* and *T. lijiangense* mycorrhization of *C. rockii* may shape the bacterial community structure and diversity in the mycorrhizosphere. The expected results will provide valuable information in improving field cultivation of these two ecologically and economically important *Tuber* species.

## Materials and methods

### Seedling cultivation and ectomycorrhizal inoculation

Seeds of *C. rockii* were collected from the Kunming Botanical Garden (KIB). Before germination, seeds were soaked in tap water for one day, with an initial water temperature of 55°C (Mao et al., 2013), then surface sterilized in sodium hypochlorite (2% available chlorine) for 2 h. After being thoroughly rinsed in distilled water, seeds were germinated in a large plastic crate, which was lined with a cotton mesh that held a sterilized growth substrate (perlite: vermiculite: water at 1:1:1), in a greenhouse at KIB, under natural conditions.

Fresh ascomata of *T. indicum* and *T. lijiangense* were purchased from local markets in Kunming, Yunnan, China. After being identified by both morphological and molecular techniques, these ascomata were then sliced and dried at room temperature for over 72 h and stored in plastic boxes at 4°C until use. The synthesis of ECM seedlings was performed according to our previous study (Huang et al., 2022). In brief, 3-month-old seedlings of similar size were selected, washed and transplanted into sterilized substrate (peat: vermiculite: perlite: water at 2:3:1:1) at pH 7.3, adjusted with calcium carbonate (0.19 g/L) and magnesium carbonate (0.1 g/L). The inocula of *T. indicum* and *T. lijiangense* were obtained by blending the ascomata, which had been soaked in non-sterile distilled water for 24 h at 4°C. Each seedling was inoculated with 10 mL spore slurry (~5 ×10^6^ spores) in May 2020. Meanwhile, each uninoculated control seedling received 10 mL of sterilized spore slurry. Seedlings were grown in 688-ml square plastic pots (13.2 × 6.4 × 9.1 cm), with one seedling per pot. A total of 38 C*. rockii* seedlings were grown in a random arrangement of pots in a greenhouse at KIB. Of them, 16 were inoculated with *T. indicum*, 16 with *T. lijiangense*, and 6 were the control group. Seedlings were watered two or three times per week with tap water. No fertilizer was added during the whole experiment.

### Ectomycorrhizal analyses

The macro-morphological characters of *T. indicum* and *T. lijiangense* mycorrhizae associated with the seedlings were examined and photographed 6 months after inoculation under a stereomicroscope (Leica S8AP0, Leica Microsytems, Wetzlar, Germany). To confirm fungal identities, 5 colonized root tips from each seedling were collected for DNA extraction. The Internal Transcribed Spacer (ITS) region of the ribosomal DNA was amplified with the ITS1F/ITS4 primer pair (White et al., 1990; Gardes and Bruns, 1993). PCR analyses were carried out on a LifeECO thermocycler (LifeBioer Technology, China) in a final volume of 25 μL containing 1μL DNA template, 1 μL of each primer (5 μM), 12.5μL of 2×Taq Mastermix, 9.5 μL ddH_2_O. The amplifications were performed with the following cycling parameters: 94°C for 5 min, followed by 35 cycles at 94°C for 1 min, 50°C for 1 min, 72°C for 1 min, and with a final extension at 72°C for 10 min. Three microliters of each PCR product were run on 1% (w/v) agarose gels and visualized by staining with ethidium bromide in a Molecular Imager Gel Doc EX system (Syngene, Shanghai, China). PCR products were Sanger sequenced in one direction by TsingKe Biological Technology, Kunming, China, and queried against published sequences deposited in GenBank database.

The mycorrhizal colonization rates were determined by following the method and criteria described by Murat (2015). Briefly, the density of mycorrhizal colonization throughout the whole root system was visually estimated and scored. All the mycorrhized (*T. indicum*: *n* = 12, *T. lijiangense*: *n* = 3) and control (*n* = 6) seedlings were used for measuring their plant growth and physiological parameters. The mycorrhizosphere substrate was used for the determination of substrate properties (*T. indicum*: *n* = 10, *T. lijiangense*: *n* = 3, control: *n* = 5), bacterial community (*T. indicum*: *n* = 5, *T. lijiangense*: *n* = 3, control: *n* = 5), and *phoC* and *phoD* gene abundance (*T. indicum*: *n* = 4, *T. lijiangense*: *n* = 3, control: *n* = 4).

### Measurement of plant growth and plant physiological parameters

A digital caliper was used to measure the following plant growth parameters: (1) plant height (from root collar to top of stem), (2) stem diameter (about 2 cm from the potting mix surface) and (3) canopy diameter (the maximal distance between the apices of opposite leaves). Numbers of leaves were simultaneously recorded.

The investigated physiological parameters of *C. rockii* seedlings included leaf photosynthetic parameters, major leaf nutrients, root superoxide dismutases (SOD) activity, and root peroxidase (POD) activity. Using a portable gas exchange fluorescence system GFS-3000 (Heinz Walz GmbH, Effeeltrich, Germany), photosynthetic rate, transpiration rate, stomatal conductance and intercellular CO_2_ concentration in the 2^nd^ fully expanded top leaf were measured between 9:30 am and 11:30 am, under 1000 μmol active radiation m^−2^ s^−1^, 400 ppm CO_2_, 25°C, and 50% relative humidity. Healthy and mature leaves in the middle of the seedling canopy were harvested to determine their fresh and dry (75°C for 48 h) biomass. The dried leaves were grounded to fine powder using a high-flux tissue mill (Scientz-48, Schneider Electric (China), Beijing, China). Leaf N was determined with a Vario MAX CN (Elementar, Langenselbold, Germany). Leaf total P, potassium (K), calcium (Ca) and manganese (Mn) were determined with an Inductively Coupled Plasma Atomic Emission Spectrometer (Thermo Jarrell Ash, MA, USA). Activity of root SOD and POD was determined according to the nitro-blue tetrazolium (NBT) (Shafi et al., 2015) and guaiacol method (Rácz et al., 2018), respectively.

### Determination of mycorrhizosphere pH, phosphatase activity and exudates

The mycorrhizosphere substrate was analyzed to determine mycorrhizosphere pH and phosphatase activity. About 4 g of fresh mycorrhizosphere substrate from each plant were carefully sampled using tweezers and spatulas, then divided into two parts: the first was stored at -20°C for DNA extraction and the second was air-dried at room temperature for pH and phosphatase activity measurements. The mycorrhizosphere pH was measured at a 1:5 ratio of substrate and water (W: V). The activities of rhizosphere acid or alkaline phosphatase (S-ACP or S-ALP) were measured separately using their respective kits (Solar-bio^®^, Beijing, China), following the manufacturer’s instructions.

After the sampling of mycorrhizosphere substrate, the entire root system with the remaining mycorrhizosphere substrate was transferred into a flask containing 150 mL 0.2 mM CaCl_2_ and 0.01 g L^-1^ Micropur (Katadyn Products, Kemptthal, Switzerland) solution to ensure cell integrity and inhibit the activity of microorganisms. Roots were gently dunked for 150 s to get rhizosphere extracts (Wang et al., 2021), which were then centrifuged (1000 rpm, 10 min), and 10 mL of the supernatant was freeze-dried for 3 days, the dried residue was re-suspended in 2 mL deionized water, centrifuged (1000 rpm, 10 min) again and filtered through a 0.45 μm membrane filter. Total organic carbon (TOC) was measured by a Nano-300 micro-spetrophotometer (ALLSHENG, Hangzhou, China) at 254 nm, with an injection volume of 1 μL (Deflandre and Gagné, 2001). The TOC concentrations were determined according to their standard curves. Organic anions were determined through a HPLC (Agilent Technologies, Tokyo, Japan) process. A UV detector (SPD-20A) monitored at 210 nm was used for the analysis of the organic anions: the injection volume was 10 μL and sample components were separated using a ZORBAX SB-Aq (4.6 × 250 mm, 5 μm) StableBond analytical column (Agilent, Delaware, USA) at 35°C column oven temperature and 10 min running time. The mobile phase was 2% acetonitrile with 0.1% H_3_PO_4_, at a flow rate of 1.0 mL min^-1^. The organic anions were identified by comparing their retention times with standards and their concentrations were determined according to their standard curves (Wang et al., 2021).

### qPCR quantification of *phoC* and *phoD* genes

Genomic DNA in 0.2 g of mycorrhizosphere substrate was extracted using a DNeasy^®^ PowerSoil^®^ Kit (QIAGEN GmbH, Hilden, Germany) following the manufacturer’s instructions. Extracted DNA was stored at -20°C until use. Substrate water content was calculated after oven-dried at 60 °C for 48 hours. The bacterial *phoC* and *phoD* genes were amplified with primers *phoC*-F (5’-CGGCTCCTATCCGTCCGG-3’), *phoC*-R (5’-CAACATCGCTTTGCCAGTG-3’) and *phoD*-F (5’-CAGTGGGACGACCACGAGGT-3’), *phoD*-R (5’-GAGGCCGATCGGCAT GTCG-3’), respectively (Fraser et al., 2017), on an ABI 7500 Real-Time PCR System (Thermo Fisher Scientific, Inc., MA, USA). All qPCRs were run in duplicated 10 μL reactions comprising 5 μL of 2x SYBR Green PCR Mix (BioRoYee, Beijing, China), 0.4 μL of each primer (0.4 μM), 1 μL of 1:20 diluted metagenomic DNA, and nuclease free sterile water. The two-step protocol for *phoC* was as follows: 3 min at 95°C, followed by 40 cycles of 10 s melt at 95°C and 30 s annealing and elongation at 58°C (Fraser et al., 2017). The three-step cycling conditions for *phoD* were as follows: 1 cycle at 94°C for 4 min, 40 cycles of 94°C for 45 s, 57°C for 30 s, 72°C for 45 s (Fraser et al., 2015). A melting step followed the amplification to ensure specificity of the reaction, from 60°C to 95°C ramping 0.1°C every second. Standard curves were prepared by 10-fold serial dilution of cloned plasmids. The copies of *phoC* and *phoD* gene were determined as per gram dry substrate based on standard curves.

### Mycorrhizosphere bacterial DNA extraction and 16S rRNA sequencing

Bacterial DNA was extracted from 0.2 g of fresh mycorrhizosphere substrate using Powersoil™ DNA isolation kits (MoBio, San Diego, CA, USA) according to the manufacturer’s instructions. The V3-V4 region of the bacterial 16S rRNA gene was amplified using the forward primer 338F (5′-ACTCCTACGGGAGGCAGCA-3′) and the reverse primer 806R (5′-GGACTACHVGGGTWTCTAAT-3′). Purified amplicons were pooled, and pair-end sequenced on an Illumina MiSeq Platform, Miseq-PE250 (Personalbio^®^, Shanghai, China). The original paired-end sequencing data were saved as the FASTQ format. The raw reads were analyzed using a QIIME2 (2019.4) software to trim off adaptors, barcodes, primers and low-quality reads. The obtained sequences with ≥ 97% similarity were merged as an operational taxonomic unit (OTU) (Edgar, 2010). The Bray-Curtis distance-based dissimilarity distance, Observed species, Simpson, Shannon and Chao1 diversity index, principal coordinate analysis (PCoA) and a Venn diagram with shared and unique OTUs were performed on a Genescloud platform of Personalbio^®^ to evaluate the bacterial community differences among non-ectomycorrhizal control, *T. indicum* and *T. lijiangense* ectomycorrhizal samples. Raw sequence data have been deposited in the NCBI Sequence Read Archive database under the bioproject identifier PRJNA854794.

### Statistical analysis

*R* software (version 3.2.3) was used for statistical analyses. Because the sample sizes are not equal across different treatments, we used unbalanced analysis of variance (ANOVA) to examine mycorrhizal inoculation effects on plant growth parameters, photosynthetic parameters, leaf nutrient concentrations, leaf water content, root POD and SOD activity, mycorrhizosphere pH, phosphatase activity, mycorrhizosphere exudates, *phoC* and *phoD* gene abundance. Data were checked for normality before analysis. Significant mycorrhizal inoculation effects were examined in more detail using Tukey honestly significant difference tests, to determine differences among *T. indicum* colonization, *T. lijiangense* colonization and un-inoculated control group. Spearman correlation analyses with Bonferroni correction were used to examine the correlations between relative abundances of the 20 most abundant OTUs and measured plant or substrate parameters including plant growth parameters such as photosynthetic parameters, leaf nutrient concentrations, leaf water content, root POD and SOD activity, mycorrhizosphere pH, phosphatase activity and mycorrhizosphere exudates. The Spearman correlation analysis was performed using genescloud tools, a free online platform for data analysis (https://www.genescloud.cn). The PCoA analysis was based on a Bray-Curtis distance at the OTU level. For all statistical analyses, a significance level of *P <* 0.05 was used.

## Results

### Ectomycorrhizae formation

**Figure 1:**
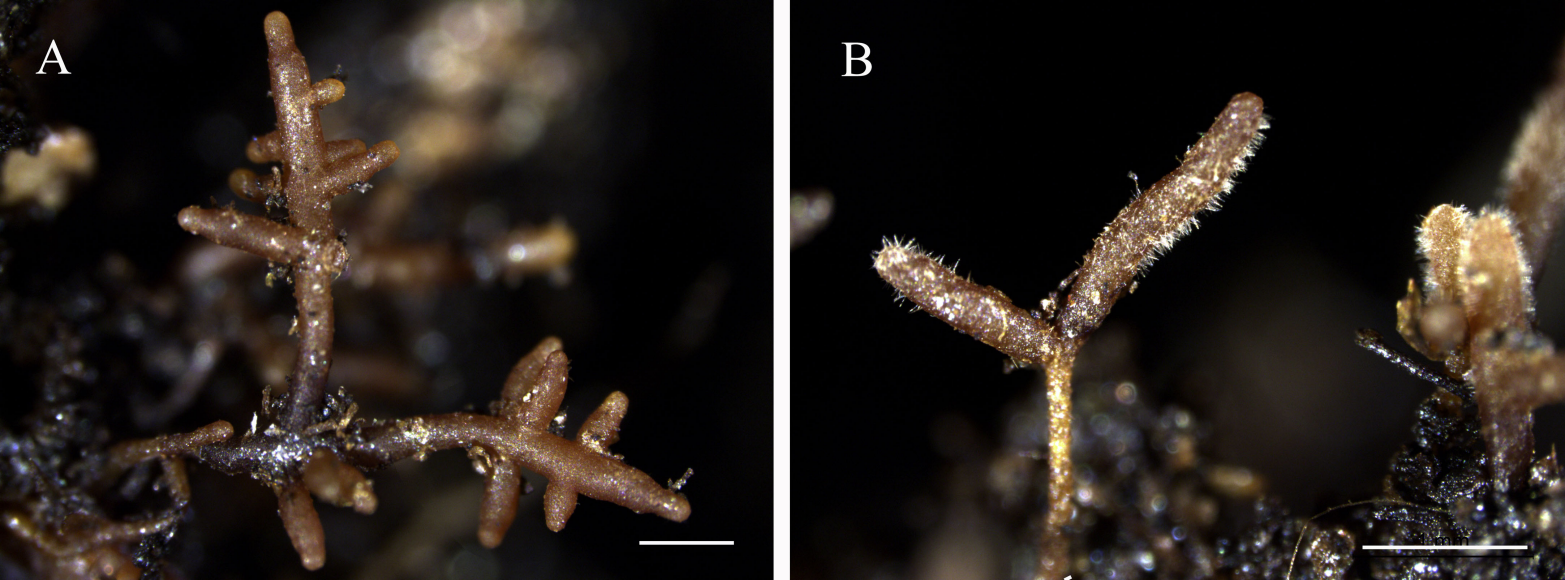
Ectomycorrhizae of *Tuber indicum* **(A)** and *T. lijiangense* **(B)** associated with *Castanopsis rockii*. (Bars = 1 mm).

Six months after inoculation, twelve out of sixteen seedlings (75%) showed *T. indicum* colonization, while only three out of sixteen seedlings (19%) showed *T. lijiangense* mycorrhiza. The ectomycorrhization was established with 50-70% colonization rates in all mycorrhizal seedlings by a rough estimation. Ectomycorrhizal root systems formed by *T. indicum* and *T. lijiangense* were simple or monopodial with lateral tips, mostly ramified in a monopodial-pinnate pattern. The mantle surface of *T. indicum* mycorrhizae was smooth to hairy or slightly woolly, with some woolly emanating hyphae, while the mantle surface of *T. lijiangense* mycorrhizae often had abundant spiky emanating hyphae (**Figure 1**). ITS sequences from *T. indicum* and *T. lijiangense* mycorrhizae formed with *C. rockii* had an identity of 99.66% and 99.83%, respectively, with published sequences of *T. indicum* (GU979058) and *T. lijiangense* (KP276188) from GenBank. The sequences of *T. lijiangense* (NO920998) and *T. indicum* (NO920999) ectomycorrhizae obtained in this study with *C. rockii* have been deposited in the GenBank. No ectomycorrhizae were found on the roots of control seedlings, and no other ectomycorrhizal fungi were detected in any of the tested seedlings.

### Effects of mycorrhization on plant growth and physiological responses

**Figure 2:**
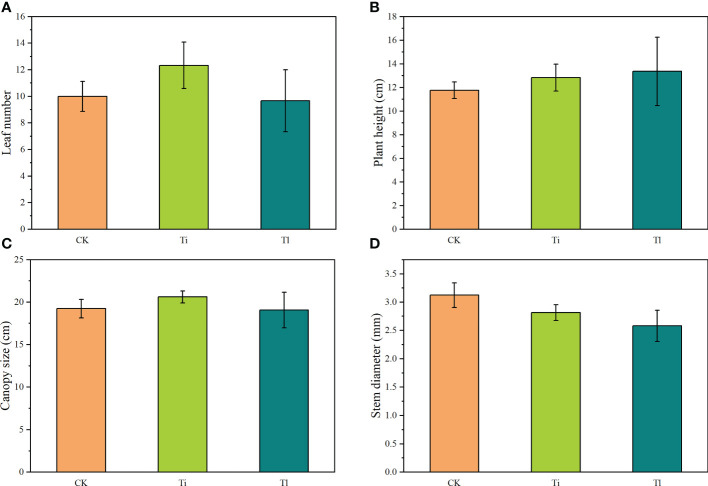
**(A–D)**. Impact of colonization by *Tuber indicum* (Ti) (*n*=12) and *T. lijiangense* (Tl) (*n*=3) on *Castanopsis rockii* seedlings regarding plant growth parameters: leaf number **(A)**, plant height **(B)**, canopy size **(C)**, and plant stem diameter **(D)**. CK = control group (*n* = 6). Bars show means ± SE.

**Figure 3:**
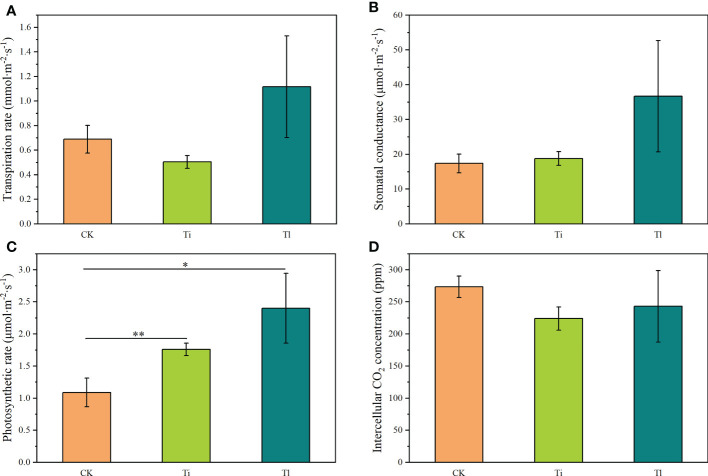
**(A–D)**. Impact of colonization by *Tuber indicum* (Ti) (*n* =12) and *T. lijiangense* (Tl) (*n*= 3) on *Castanopsis rockii* seedlings regarding leaf photosynthetic parameters: transpiration rate **(A)**, stomatal conductance **(B)**, photosynthetic rate **(C)**, intercellular CO_2_ concentration **(D)**. CK = control group (*n* = 6). Bars show means ± SE. Significance at *P*<0.05 *, and *P*<0.01 **.

**Figure 4:**
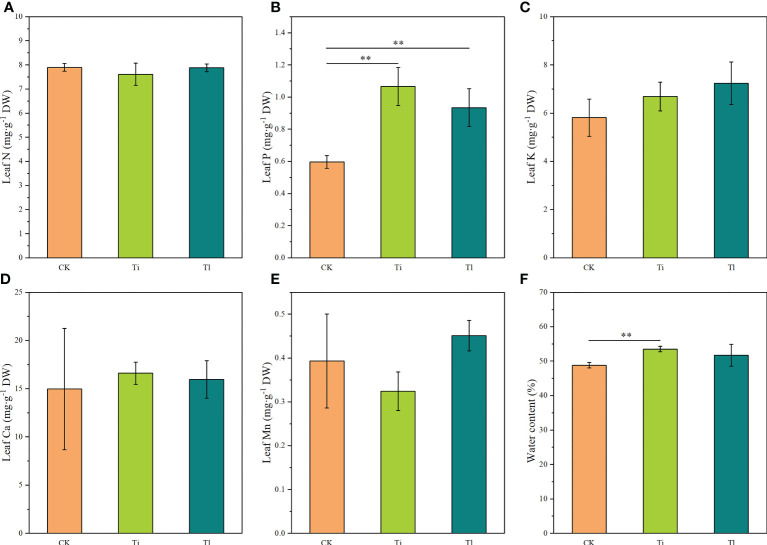
**(A–F)**. Impact of colonization by *Tuber indicum* (Ti) (*n* =11) and *T. lijiangense* (Tl) (*n* = 3) on *Castanopsis rockii* seedlings regarding plant nutrient uptake of leaf nitrogen **(A)**, leaf phosphorus **(B)**, leaf potassium **(C)**, leaf calcium **(D)**, leaf manganese **(E)**, leaf water content **(F)**. CK = control group (n = 5). Bars show means ± SE. Significance at *P*<0.01 **.

**Figure 5:**
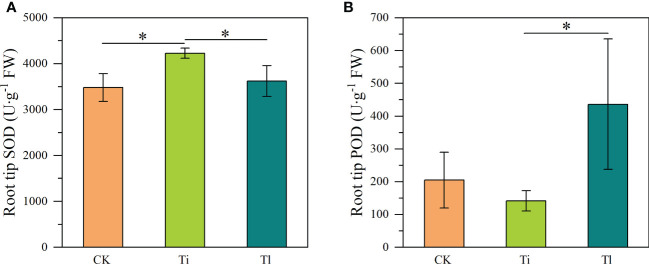
**(A, B)**. Impact of colonization by *Tuber indicum* (Ti) (*n* =10) and *T. lijiangense* (Tl) (*n* = 3) on *Castanopsis rockii* seedlings regarding root oxidase activity of superoxide dismutase SOD **(A)** and peroxidase POD **(B)**. CK = control group (*n* = 5). Bars show means ± SE. Significance at *P*<0.05 *.

Compared with uninoculated control seedlings, ECM colonization exhibited no significant effects on morphology and plant growth (**Figures 2A–D**). Leaf photosynthetic rate (*F*=7.21, *P*<0.01) (**Figure 3C**) and P concentration (*F*=5.18, *P*<0.05) (**Figure 4B**) were significantly improved by both fungi. However, transpiration rate (**Figure 3A**), leaf stomatal conductance (**Figure 3B**), intercellular CO_2_ concentration (**Figure 3D**), leaf concentrations of N (**Figure 4A**), K, Ca and Mn (**Figures 4C–E**) were not affected. Furthermore, *T. indicum* colonization significantly enhanced leaf water content (*F*=13.48, *P*<0.01), while *T. lijiangense* did not (**Figure 4F**). *T. indicum* and *T. lijiangense* colonization had opposite effects on root SOD and POD activity: *T. indicum* colonization markedly enhanced root SOD activity (*F*=4.97, *P*<0.05), while *T. lijiangense* colonization significantly reduced root POD activity (*F*=7.40, *P*<0.05) (**Figures 5A, B**). Compared to control treatment, *T. indicum* colonization significantly enhanced root SOD activity (*F*=8.54, *P*<0.05), but reduced root POD activity, while *T. lijiangense* had no significant impact on root SOD and POD activity (**Figures 5A, B**).

### Effects of mycorrhization on mycorrhizosphere pH, phosphatase activity and exudates

**Figure 6:**
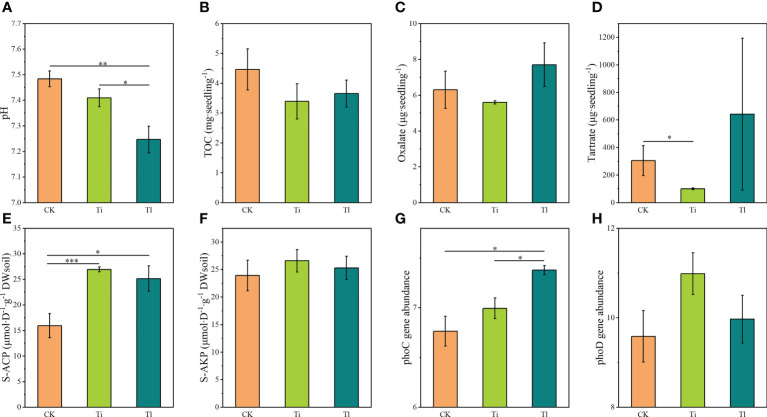
**(A–H)**. Impact of colonization by *Tuber indicum* (Ti) and *T. lijiangense* (Tl) on *Castanopsis rockii* seedlings regarding mycorrhizosphere characterstics: pH **(A)**, total organic carbon **(B)**, oxalate **(C)**, tartrate **(D)**, acid phosphatase activity **(E)**, alkaline phosphatase activity **(F)**. For these parameters the sample size was as follows: Ti =10, Tl = 3, whereas for *phoC* gene abundance **(G)**, and *phoD* gene abundance **(H)** it was Ti = 4, Tl = 3. CK = control group (*n* = 4). Bars show means ± SE. Significance at *P*<0.05 *, *P*<0.01 ** and *P*<0.001 ***.

Seedlings associated with *T. lijiangense* had significantly lower mycorrhizosphere pH than control seedlings (*F*=17.71, *P*<0.01) and *T. indicum* colonized seedlings (*F*=5.10, *P*<0.05) (**Figure 6A**). In comparison with control group, *T. indicum* colonization significantly reduced tartrate accumulation (*F*=6.82, *P*<0.05) (**Figure 6D**). On the other hand, mycorrhizal colonization with both *Tuber* species did not significantly reduce mycorrhizosphere TOC (**Figure 6B**), and oxalate content (**Figure 6C**).

Ectomycorrhizal colonization significantly improved mycorrhizosphere S-ACP activity of seedlings mycorrhized with both *T. indicum* (*F*=18.43, *P*<0.001) and *T. lijiangense* (*F*=6.41, *P*<0.05) in comparison with control group (**Figure 6E**), whereas S-ALP activity did not change (**Figures 6F**). *T. lijiangense* colonization significantly increased bacterial *phoC* gene abundance in comparison with *T. indicum* colonization (*F*=8.79, *P*<0.05) and the control (*F*=11.64, *P*<0.05), while *T. indicum* did not have any significant impact on this parameter (**Figure 6G**). The abundance of *phoD* gene was not affected by the colonization of both *Tuber* species (**Figure 6H**).

### Effects of mycorrhization on mycorrhizosphere bacterial communities

Between 76,889 and 101,639 bacterial 16S rRNA gene reads were obtained from Illumina Paired-end sequencing for each sample after quality control procedures generated a total of 18,112 bacterial operational taxonomic units (OTUs) for further analysis. These bacterial OTUs were clustered into 35 phyla, 101 classes, 255 orders, 443 families, 855 genera and 1961 species. At the phylum level, Proteobacteria, Chloroflexi and Acidobacteria were the dominant bacterial phylum, with relative abundances of 47.9%, 15.9% and 14.9%, respectively. The relative abundance of Proteobacteria was 50.1%, 46.9% and 45.9% in the sample of plants colonized by *T. indicum*, *T. lijiangense* and control group, respectively.

**Figure 7:**
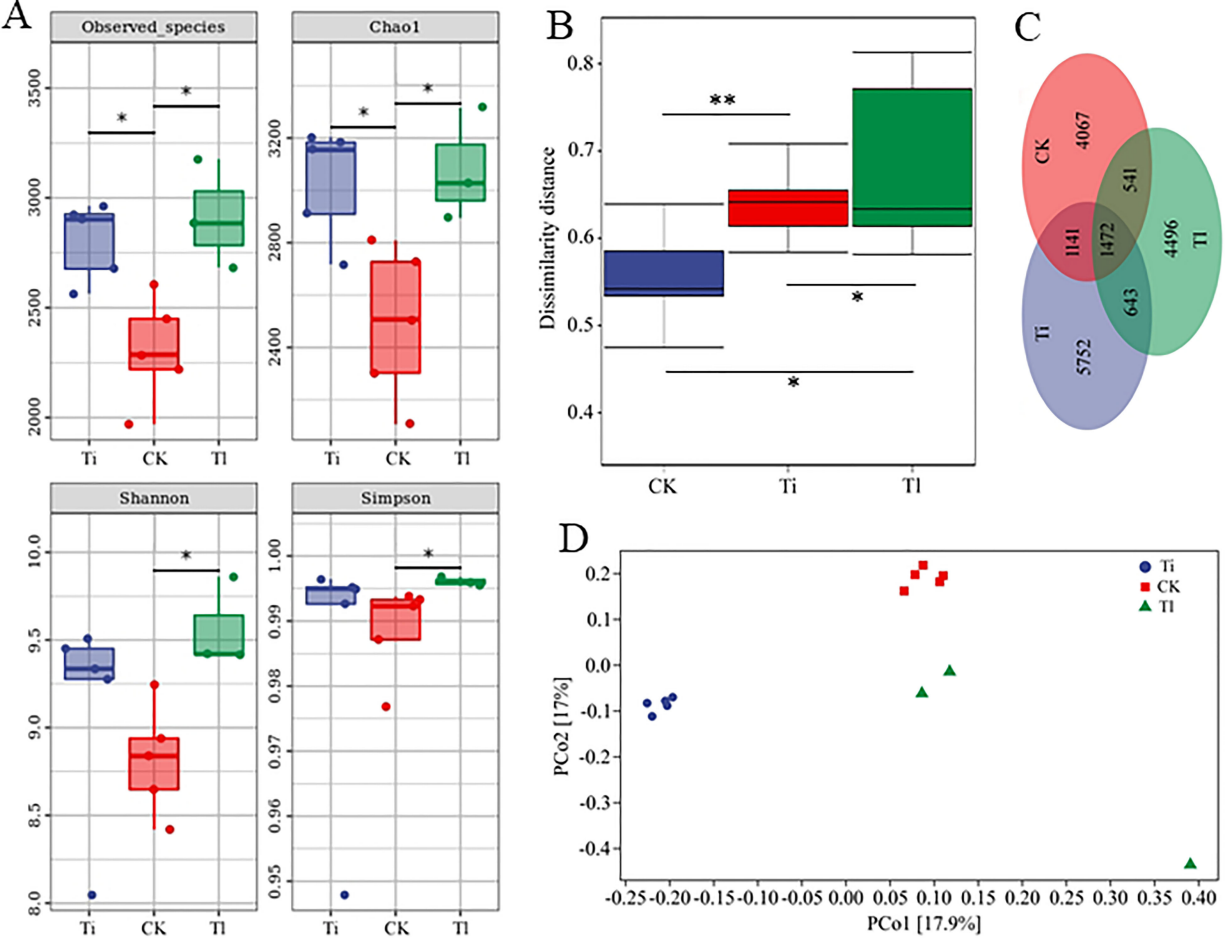
**(A–D)**. Impact of colonization by *Tuber indicum* (Ti) (*n* =5) and *T. lijiangense* (Tl) (*n* = 3) on *Castanopsis rockii* seedlings regarding bacterial community richness and diversity **(A)**: Observed species, Chao1 diversity, Shannon index and Simpson index, dissimilarity distance **(B)**, Venn figure showing unique ad operation taxonomic units (OTUs) **(C)**, and principal coordinate analysis (PcoA) **(D)**. CK = control group (*n* = 5). Significance at *P*<0.05 *, and *P*<0.01 **.

**Figure 8:**
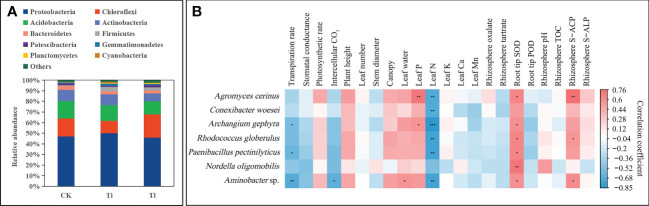
**(A, B)**. Impact of colonization by *Tuber indicum* (Ti) (*n* =5) and *T. lijiangense* (Tl) (*n* = 3) on *Castanopsis rockii* seedlings regarding bacterial community structure **(A)**. Heatmap showed the results of Spearman correlation analysis with Bonferroni correction between the 20 most prominent bacterial taxa and plant and soil parameters **(B)**. CK = control group (*n* = 5). Significance at *P*<0.05 *, *P*<0.01 ** and *P*<0.001 ***.

The analyses of bacterial community indicated that *T. lijiangense* colonization showed significant differences regarding Observed OTU richness, Chao 1, Shannon and Simpson index (α-diversity, **Figure 7A**) compared with the control group, whereas *T. indicum* colonization showed significant differences only in Observed OTU richness and Chao 1 index. No significant differences were found between *T. lijiangense* and *T. indicum* colonization regarding all the α-diversity index (**Figure 7A**). Furthermore, bacterial communities in the mycorrhizosphere of *C. rockii* plants colonized by *T. indicum* and *T. lijiangense* revealed significant differences between them and in comparison with the control group regarding β-diversity (dissimilarity distance, **Figure 7B** total of 18,112 OTUs were obtained, with only 8% of them being shared among *T. indicum*, *T. lijiangense* and the control group (**Figure 7C**). In addition, *T. indicum* and *T. lijiangense* mycorrhizal samples and control group samples were clearly separated by PCoA (**Figure 7D**) Mycorrhizosphere communities of plants inoculated with *T. indicum* showed a significantly higher relative abundance of Firmicutes than control plants (*F*=14.34, *P*<0.01), and showed significantly less relative abundance of Chloroflexi than colonization by *T. lijiangense* (*F*=14.26, *P*<0.01) (**Figure 8A**).

The 20 most abundant OTUs were selected to perform the Spearman correlation analysis with the experimental plants and soil parameters with the exception of *phoC* and *phoD* because the samples used for bacterial analysis were not the same ones as for *phoC* and *phoD* gene analysis. Only 7 OTUs (Actinobacteria: *Agromyces cerinus*, *Conexibacter woesei* and *Rhodococcus globerulus*; Proteobacteria: *Aminobacter* sp., *Archangium gephyra*, and *Nordella oligomobilis*; Firmicutes: *Paenibacillus pectinilyticus*) showed significant relationships with the selected parameters (**Figure 8B**). Noteworthy, Leaf P was positively correlated with *Ag. cerinus* and *Ar. gephyra*. Rhizosphere S-ACP activity was positively correlated with *Ag. cerinus*, *R. globerulus* and *Aminobacter* sp. (**Figure 8B**).

## Discussion

### Effects of plant growth and physiological parameters

This work represents the first report of the successful mycorrhizal associations between two *Tuber* species (*T. indicum* and *T. lijiangense*) and *C. rockii* seedlings. The most important morphological character that allows to distinguish the ectomycorrhizae of the two selected *Tuber* species is the presence and the shape of the emanating hyphae. *T. indicum* generally shows woolly or absence of emanating hyphae while *T. lijiangense* shows abundant spiky emanating hyphae, consistent with previous studies (García-Montero et al., 2008; Wan et al., 2016). The colonization rate of both *Tuber* species was 50-70% by roughly estimates, but only three out of sixteen seedlings inoculated with *T. lijiangense* formed mycorrhizae. This different colonization patterns caused strongly different sample sizes. Soil physicochemical properties are an important factor affecting mycorrhizae formation (Bustan et al., 2006; Geng et al., 2009). Considering the fact that the optimal pH for mycelial growth of *T. japonicum* (white truffle) was 5-6 (Nakano et al, 2020), suggests that a slightly lower initial pH of the substrate might be more suitable for *T. lijiangense*.

Both fungi significantly enhanced photosynthesis and leaf P concentration of *C. rockii*, which is consistent with an enhanced photosynthesis and P acquisition in ectomycorrhized *Pinus* spp. (Reid et al., 1983; Choi et al., 2005), *Eucalyptus camaldulensis* (Dixon and Hiol-Hiol, 1992) and *Quercus* spp. (Nehls and Plassard, 2018; Wang et al., 2021; Huang et al., 2022). The enhanced photosynthetic rate could lead to an enhancement of C assimilates, and thus plant growth. The photosynthesis-assimilated C might have been allocated to root exudates and fungal tissues (Wang et al., 2021). However, our results did not agree with this trend because it was found that an enhanced photosynthetic rate and P uptake did not significantly increased plant growth.

### Effects of mycorrhization on mycorrhizosphere

We observed lower content of organic anions in the mycorrhizosphere of *C. rockii* seedlings in association with *T. indicum* compared to those with *T. lijiangense* and control plants, with the release of tartrate being especially low in case of *T. indicum* colonization. The pH in the mycorrhizosphere of seedlings inoculated with *T. lijiangense* was lower than in those inoculated with *T. indicum* colonization. These colonization is in accordance with the findings by Casarin et al. (2004), who found that mycorrhizosphere pH is reduced by the release of organic anions. It has been reported that ECM colonization decreases root released organic anions (van Scholl et al., 2006; Meier et al., 2013; Wang et al., 2021), although ECM species such as *Paxillus involutus* and *Piloderma croceum* increase rhizosphere oxalate exudation, suggesting that the exudation of organic anions depends on fungal taxon (van Scholl et al., 2006). Nakano et al. (2020) demonstrated that the optimal pH for *T. japonicum* (a white truffle as *T. lijiangense*) mycelial growth was much lower than that of *T. himalayense* and *T. longispinosum* (both black truffles). Whether this trend will be confirmed is a matter of future investigations. The fact that *T. lijiangese* (white truffle) is capable of lowering rhizopshere pH supports the hypothesis that ECM fungi modify soil property to their benefit (García-Montero et al., 2017).

Phosphorus is a limiting nutrient in many environments, but plants and microbes have evolved various mechanisms for acquiring soil P, including excretion of phosphatase enzymes (Fraser et al., 2017). In this study, we found that these two *Tuber* species could significantly favor rhizosphere *phoC* abundance and acid phosphatase activity in the rhizosphere of *C. rockii* seedlings, combined with an improved leaf P concentration. Ectomycorrhizal fungi regulate *phoC*-harbouring microbes to mobilize soil organic P to improved plant P acquisition (Ragot et al., 2015; Fraser et al., 2017). However, neither of the two species studied had any effect on rhizosphere *phoD* abundance and alkaline phosphatase activity, in agreement with the reduction of species diversity and richness of *phoD*-harbouring bacteria in rhizosphere of *P. armandii* seedlings colonized by *T. melanosporum* (Zhang et al., 2020).

It is well known that SOD and POD play an important anti-oxidation and anti-stress physiological function in plant (Bela et al., 2015). Zhang et al. (2019) found *T. indicum* inoculation improved the SOD activity of *Q. acutissima* roots but had no obvious effects on the host plant POD activity, which is consistent with our findings on *C. rockii*. Similar effects on physiological responses of host plants colonized by *T. indicum* and *T. melanosporum* have been reported (Zhang et al., 2020). In our study, *T. indicum* and *T. lijiangense* showed different influences on root SOD and POD activity.

### Effects of mycorrhization on mycorrhizosphere bacterial community

It has been shown that bacterial richness and diversity in the mycorrhizospehere of *T. indicum* or *T. panzhihuanense* and *T. borchii* are significantly higher than those in the non-ectomycorrhizal roots (Li et al., 2018; Li et al., 2019; Yang et al., 2019). In our study, we found that both *Tuber* species colonization significantly increased the richness (Chao1 and Observed OTU richness) of bacterial communities over the control, while *T. lijiangense* colonization also significantly enriched the diversity (Shannon and Simpson) of bacterial communities. It was reported that the presence of ectomycorrhizal fungi can alter the soil microbial composition (Li et al., 2018; Wang et al., 2021). In the present study, the microbial community composition in *T. indicum*, *T. lijiangense* and control samples show significant differences, revealing that different *Tuber* species differently shaped bacterial communities. This finding is most likely caused by different fungus-host combinations that provide different rhizosphere niches (Beckers et al., 2017).

Furthermore, we found that Proteobacteria was the dominant phylum in the mycorrhizosphere of both inoculated seedlings and control group, in agreement with results from other studies (Deveau et al., 2016; Wang et al., 2021). The phylum Chloroflexi was significantly enriched by *T. lijiangense* mycorrhization, while *T. indicum* colonization significantly enriched Firmicutes in comparison with the control. Some soil Firmicutes have a strong ability to resists dehydration and extreme conditions (Teixeira et al., 2010; Bulgarelli et al., 2013; Trivedi et al., 2020), and Chloroflexi have been reported having nitrification and carbon cycling abilities (Hug et al., 2013; Chu et al., 2015), which could influence the resistance to stress and N nutrition uptake of plant.

*Ag. cerinus* has been shown to be potassium-solubilizing (Yang et al., 2017). In our study, *Ag. cerinus* had significantly positive correlation with mycorrhizosphere S-ACP and leaf P concentration, which might imply that this bacterium also has a role in P mobilization.

## Conclusions

This study showed that *T. indicum* and *T. lijiangese* have different colonization patterns with *C. rockii*, and they differentially regulate host plant physiological responses and mycorrhizosphere bacterial communities. However, it should be noted that the unbalanced sample size from *T. indiucm*, *T. lijiangense* and control group might limit our results, and additional studies using balanced sample are thus needed to reinforce these findings. In our study, mycorrhization with both fungi significantly enhanced leaf photosynthetic rate, promoted P nutrient acquisition and altered mycorrhizosphere bacterial community of host seedlings. Relative abundances of *Ag. cerinus* positively correlated to mycorrhizosphere acid phosphatase activity and leaf P concentration, and might thus play an important role in soil P mobilization. These results indicate that ECM fungi species can regulate P-cycling and bacterial community structure in the mycorrhizosphere. However, different *Tuber* species had different influences on mycorrhizosphere organic anions, root SOD and POD activity, as well as bacterial communities. The content of rhizosphere organic anions caused by *T. lijiangense* mycorrhization was higher than that in *T. indicum*, and *T. indicum* colonization caused significantly higher root SOD but significantly lower root POD than *T. lijiangense* colonization. These results provide insights into a better understanding of P mobilization and utilization of mycorrhizal seedlings, as well as a theoretical basis for field cultivation of truffles. However, results from this present study are from greenhouse conditions. Whether these two ECM fungi have similar effects on field plants needs further explorations.

## Data availability statement

The datasets presented in this study can be found in online repositories. The names of the repository/repositories and accession number(s) can be found in the article/supplementary material.

## Author contributions

LH carried out the experimentation, data collection, partial data analysis, and wrote the manuscript. YW and YL analyzed data, conceptualized and reviewed the manuscript. YW and FY designed and instructed this study. JY helped with *phoC* and *phoD* analysis. SW contributed to ECM fungi materials and reviewed the manuscript, XH, CC, YL and XS revised the manuscript. All authors contributed to the article and approved the submitted version.
